# *Delftia* spp as Opportunistic Pathogens: a narrative review

**DOI:** 10.1016/j.nmni.2026.101705

**Published:** 2026-01-16

**Authors:** Michael P. Ryan, J. Tony Pembroke

**Affiliations:** a Department of Chemical Sciences, University of Limerick, Limerick, V94 T9PX, Ireland; b Bernal Institute, University of Limerick, Limerick, V94 T9PX, Ireland

**Keywords:** *Delftia*, Nosocomial infection, Opportunistic pathogen

## Abstract

Non-fermenting Gram-negative bacteria pose a considerable challenge in medical settings and are increasingly implicated in infections in these settings. Many are opportunistic pathogens that primarily affect patients with other acute or chronic health conditions. Among them, Delftia species—particularly *Delftia acidovorans* - have traditionally been regarded as of limited clinical relevance. However, a comprehensive literature review has identified 175 reported cases of Delftia infections, with *D. acidovorans* accounting the majority cases (87.4 %). Bacteraemia was the most commonly associated condition, reported in 23 cases (13.1 %) with other infections such as pneumonia (9.8 %), sepsis (3.4 %) and peritonitis (2.9 %) also being prominent. The findings suggested that the antibiotics ceftazidime, ciprofloxacin and imipenem are usually effective in treating Delftia infections, but that gentamicin should be avoided. These findings suggest that while *Delftia* spp. may not be a widespread pathogen awareness and appropriate diagnostic recognition are required.

## 1 Introduction

The emergence of Gram-negative, non-fermenting bacteria as a common cause of infections in immunocompromised patients has become a major issue in clinical settings. These organisms are ubiquitous in natural environment being found in many different niches [1]. These niches include soil, plants and animals [2] and various water sources (including hospital water, aircraft water, bottled drinking water, purified water) [[3], [4], [5], [6]].

These bacteria are frequently resistant to many different antimicrobials. Examples include resistance to penicillin's, aminoglycosides and monobactam's in *R. pickettii* [7] and β -lactams in *Ochrobactrum* spp [8].

While the major pathogenic bacteria of this heterogeneous group (*Pseudomonas aeruginosa* [9]*,* the *Burkholderia cepacian* complex [10], *Acinetobacter baumannii* [11]) are well known other lesser-known bacteria are also emerging as pathogenic organisms. These include bacterial species such as*, Ralstonia pickettii* [12], *Sphingomonas paucimobilis* [13]*, Ochrobactrum* spp [14]*, Comanonas* spp [15] and *Brevundimonas* spp [16].

*Delftia* spp. are one of these emerging genera. They have been isolated from many different environmental niches, including water sources [17], aircraft water [4], wastewater [18], soil, plants [19], and animals [20]. *Delftia* spp. have been shown to degrade xenobiotic pollutants [21,22] and to detoxify heavy metals [23,24] this includes the biomineralize of gold. *Delftia* spp. are believed to be of low virulence and routine surveillance systems rarely track Delftia explicitly. Despite this *Delftia* spp have caused infections, including serious infection such as endocarditis [25], sepsis [26] and pneumonia [27] in immunocompetent hosts.

Examination of literature sources (both scientific and medical) established that *Delftia* spp give rise to a multitude of different infections potentially indicating a stronger pathogenic potential than was supposed. The overall aim of this study was to support the thesis that *Delftia* spp are important pathogens needing more attention.

### 1.1 Genus Delftia

**Fig. 1:**
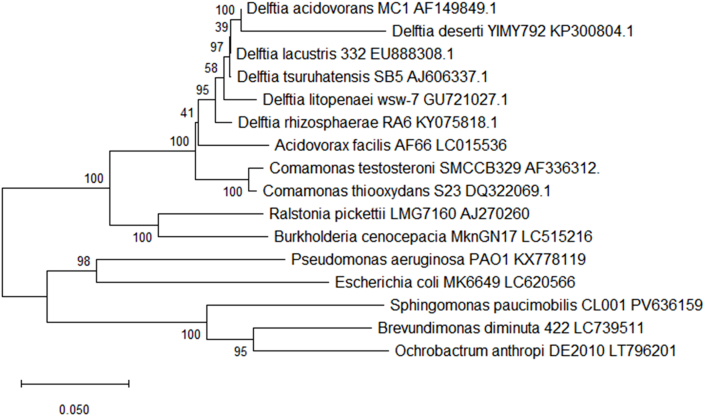
Phylogenetic tree of the genus *Delftia* built with 16S rDNA genes (partial sequences of ∼1400 bp) (gene accession numbers are given along with the species name) with the other Pseudomonadota species. The tree was built with neighbour-joining (Tajma-Nei method) using the MEGA 11 software package. Bootstrap values are represented by numbers at nodes. These are based on 500 resamplings. Bar, 0.0050 substitutions per site [59]. It should be noted that the above tree is based upon 16S rDNA and, as such, is suggestive only.

The Delftia genus was first described in 1999 with the reassignment of *Comamonas acidovorans* to Delftia [28]. Five further species were added to the genus over the next 20 years: *Delftia tsuruhatensis* in 2003 [29]*, Delftia lacustris* in 2009 [30]*, Delftia litopenaei* in 2012 [31]*, Delftia deserti* in 2015 [32] and *Delftia rhizosphaerae* in 2017 [33] (see Fig. 1). Delftia is a betaproteobacterial genus widely spread in the environment, these niches include soil, water sources, plants, and both healthy and diseased animal. The genus has a wide array of potential applications both in agriculture and in industry. These applications include immobilization of heavy metals including lead [34]and zinc [35], enhancement of plant growth [36] and microbial treatment of hydrocarbon-polluted soils [37]. *Delftia acidovorans* is the type species of the genus with the type strain being ATCC 15668.

### 1.2 Genomics of Delftia

Bhat et al. carried out a pangenomic analysis of 61 Delftia species genomes. The genomes studied represented three of the described Delftia species, *D. acidovorans, D. tsuruhatensis* and *D. lacustris* with an additional 27 unassigned Delftia species (*D. litopenaei*, *D. rhizosphaerae* and *D. deserti* have no genome data) [38].

Median genome size for these 61 strains was 6.6 Mbp, with the largest size being 7.3Mbp [38]. These results agree with the multiple genome analysis of *D. tsuruhatensis* [39]. Phylogenomic comparison of these 61 Delftia genomes indicated that the genus can be split into two clades, the first named the *Delftia acidovorans* clade contains isolates from soil and plant rhizospheres, while the second named the *Delftia lacustris/Delftia tsuruhatensis* clade contains isolates from humans and sludge [38]. The ecological versatility of the genus was supported by the high pan-genome size observed, ranging from 17 986 genes for *D acidovorans* clade and 18 987 genes for clade *D. lacustris/D. tsuruhatensis* clade [38]. Although both clade members showed ecological specialism the proteomes were quite similar with only 27 protein differences present in the *D acidovorans* clade and 12 proteins present in the *D. lacustris*/*D. tsuruhatensis* clade not in the first clade [38]. Yin et al. (2022) also carried out gene synteny analysis amongst 2 key species of *D. tsuruhatensis*, strains CM13 and TR1180. This showed a high level of synteny with many rearrangements and inversions amongst the comparison and some 37 synteny blocks recognised, ranging from 6,735,844 bp (93.6 %) in CM13 and 6,606,877 bp (98.4 %) in TR1180 suggestive of rearrangements and inversions occurred during the genomic evolution of *D. tsuruhatensis* [39].

### 1.3 Mobile genetic elements

Genome analysis within the comparison revealed many types of mobile elements associated with *D. tsuruhatensis*, including insertion sequenced, genomic islands and prophage with multiple types observed of each element and in some instances, calculations suggesting they composed up to 10 % of the larger species genome. CRISPR loci, that defend the species against mobile element and prophage invasion, were also reported. Yin et al. concluded that such mobile elements contributed to the genomic diversity of *D. tsuruhatensis* [39].

### 1.4 Virulence factors

Macromolecular secretion systems, capable of DNA and protein secretion and often associated with virulence were also discovered during the pan genome analysis including type I (T1SS), II (T2SS), IV (T4SS), VI (T6SS), IV (T4P), flagellum and Tad pilus secretion systems. Although their roles in *D. tsuruhatensis* have yet to be identified, many may play a role in pathogenicity [39]. Analysis of putative virulence genes amongst the genome comparison revealed 112 gene families, matched with virulence genes in the PHI-base database, some 80 of which were present in most of the genomes. The genes identified were predicted to be related with determining nosocomial infections, such as urinary tract infection, meningococcal infection, gastric infections, bloodstream infection, skin infection, and prosthetic joint infection, supporting the proposition of *D. tsuruhatensis* as an emerging opportunistic pathogen particularly in immunocompromised hosts [39]. Another study undertaken out by Andriyanov et al., identified multiple virulence factors homologs in the genomes of *Delftia* spp. Each strain was found to have 24 to 31 putative virulence factors genes. These genes were involved in motility, adherence, immune modulation, stress survival, secretion systems, siderophores and general cellular fitness [40]. *Delftia acidovorans* has been found to form biofilm that are resistance to Chlorhexidine [41,42].

## 2 Methodology

All accessible publications (journal articles, medical case reports and conference proceedings) discussing *Delftia* spp infections were recovered using PubMed, Web of Knowledge and Google Scholar. The terms ‘*Delftia’*, ‘*Delftia’* spp.’ ‘*Comamonas acidovorans’*, ‘*Pseudomonas acidovorans’* (previous names of *Delftia acidovorans*) and Delftia species recorded in Table 1 were all investigated from January 1976 until January 2026. Any publications that examined human infection were reviewed and the desired information extracted from them. The information (where available) obtained included year, geographic location, patient information (age, sex, any underlying medical condition[s]), antimicrobial susceptibility, treatment (antibiotics, catheter removal, etc) and patient outcomes (recovery, death, etc.). This was used to populate Table 2, Table 3, Table 4, Table 5. The references cited in these publications were also checked for any infection reports that were not found in the database searches.

**Table 1:** Listing of validly published Delftia species.

Species	Isolation site	Origin	Genome sequences	Reference
*Delftia acidovorans*	The Netherlands	Soil enriched with acetamide	Strain: SPH-1,	Wen et al., 1999 [28]
			Size: 6.767 Mb	
			Ref Genome: CP000884.1 (47 genomes)	
*Delftia deserti*	China	Desert soil sample	Strain: KCTC42377,	Li et al., 2015 [32]
			Size: 7 Mb	
			Ref Genome: JBHUIG000000000	
*Delftia lacustris*	Denmark	Fresh water lake	Strain: LZ-C	Jørgensen et al., 2009 [30]
			Size: 7 Mb	
			Ref Genome: GCF_001017795.1 (9 genomes)	
*Delftia litopenaei*	Taiwan/	Fresh water shrimp pond	N/A	Chen et al., 2012 [31]
*Delftia rhizosphaerae*	Spain	Rhizosphere of *Cistus ladanifer* plant	N/A	Carro et al., 2017 [33]
*Delftia tsuruhatensis*	Japan	Activated.	Strain: 391	Shigematsu et al., 2003 [29]
			Size: 6.7 Mb	
			Ref Genome: GCF_001017795.1 (9 genomes)

**Table 2:** Incidences of *D. acidovorans* infection from 1976 to 2025– Main characteristics of the case reports.

Author (Ref)	Year	Location	Age/Sex	Co-morbidity	Type of infection	Antibiotic Resistance	Antibiotic Susceptibility	Treatment	Patient outcome
Weinstein et al., [53]	1976	USA	N/A	N/A	Bacteremia	N/A	N/A	N/A	Complete recovery
Brinser and Torczynski [60]	1977	USA	60/F	None	Corneal ulcers	Ampicillin, Bacitracin, Carbenicillin Cephalothin, Penicillin	Colistin, Gentamicin, Neomycin, Polymyxin B Tetracycline sulfonamide,	Carbenicillin Gentamicin Along with topically applied gentamicin, colymycin, and 1 % atropine.	Complete recovery
Horowitz, et al., [25]	1989	USA	42/F	Intravenous drug user and alcohol abuse	Endocarditis	Amikacin, Ampicillin, Cefazolin, Cefuroxime, Gentamicin, Ticarcillin, Tobramycin,	Aztreonam, Cefoxitin, Cefoperazon, Ceftazidime, Cefotaxime, Ceftriaxone, Chloramphenicol, Ciprofloxacin Mezlocillin, Piperacillin, SXT	Penicillin, Ceftazidime, oral ciprofloxacin, Vancomycin and amphotericin B	Death
Reina et al., [61]	1991	Spain	7/M	None	Suppurative Otitis	Ampicillin, Cefazolin, Cefuroxime, Carbenicillin, Gentamicin	Amikacin, Aztreonam Cefotaxime, Ceftazidime, Ceftriaxone, Chloramphenicol, Ciprofloxacin, Imipenem, Tobramycin, SXT	Ceftazidime	Complete recovery
Stonecipher et al., [62]	1991	USA	48/F	None	Ocular Infection	Ampicillin, Cefazolin, Gentamicin	Tobramycin	Gentamicin Followed by Tobramycin	Complete recovery
Stonecipher et al., [62]	1991	USA	57/F	None	Ocular Infection	N/A	N/A	prednisolone acetate eyedrops	Complete recovery
Stonecipher et al., [62]	1991	USA	76/F	None	Ocular Infection	N/A	N/A	Oral doxycycline and topical erythromycin Followed by Polymixin B	Complete recovery
Stonecipher et al., [62]	1991	USA	16/F	None *Acinetobacter calcoaceticus*	Ocular Infection	N/A	Gentamicin sulfate	Gentamicin sulfate	Complete recovery
Stonecipher et al., [62]	1991	USA	32/M	None *Bacillus cereus, Flavobacterium* spp*. Acanthamoeba*	Ocular Infection	N/A	N/A	Propamidine, gramicidin, Scopolamine hydrobromide	Complete recovery
Stonecipher et al., [62]	1991	USA	26/W	None *Acanthamoeba*	Ocular Infection	N/A	N/A	N/A	Complete recovery
Castagnola, et al., [63]	1994	Italy	9/M	Non-Hodgkin's Lymphoma	Central venous catheter related infection	Broad spectrum penicillin's, Gentamicin	Arninoglycosides, Carbapencrns, Cephalosporins, Monobactam, Quinolones, SXT,	Ceftazidime, Vancomycin. Changed to Amikacin. Removal of catheter.	Complete recovery
Ender, et al., [64]	1994	unknown	4/F	Metastatic neuroblastoma	Vascular Catheter related Bactereamia	All aminoglycosides	Aztreonam, Cephalosporins, Ciprofloxacin, Piperacillin, Ticarcillan	Ceftazidime therapy, intravenous ciprofloxacin, ceftriaxone, cephalosporin and the catheter was removed	Complete recovery
Lair, et al., [65]	1996	France	27/M	HIV/AIDS *Oerskovia turbata*	Bactereamia associated with a central venous catheter	Amikacin, Cefsulodin, Colimycin, Gentamicin, Ticarcillin	Ceftazidime, Imipenem Piperacillin, Pefloxacin	Catheter removed, Imipenem (500 mg twice daily) and Amikacin (400 mg)	Complete recovery
López-Menchero, et al., [66]	1998	Spain	35/F	End-stage renal disease secondary to chronic glomerulonephritis, with a single kidney, was included in the CAPD	Peritonits	N/A	N/A	Oral ciprofloxacin (750 mg/12 h for 14 days), 1 g ceftazidime IP daily. piperacillin to the treatment (4 g/12 h, intravenous). Peritoneal catheter was removed.	Complete recovery
Ojeda-Vargas, et al., [67]	1999	Gran Canaria	61/M	Hemiplegia	Urinary tract infection	Amikacin, Ampicillim, Fosfomicin, Tobramycin	Aztreonam, Cefotaxime, Ciprofloxacin, Imipenem, Norfloxacin, Ticarcillin, SXT	Two-week course of Norfloxacin (400 mg orally twice daily)	Complete recovery
Perla & Knutson [68],	2005	USA	35/M	Intravenous drug abuser	Bactereamia associated with a contaminated needle	Gentamicin	Ceftazidime, Cefotaxime, Ceftriaxone, Imipenem, Levofloxacin, Piperacillin -Tazobactum, SXT	10 days of Levofloxacin	Complete recovery
Oliver, et al., [69]	2005	USA	30/M	Healthy male went to hospital after receiving a gunshot wound *Ochrabactrum anthropi*	Bactereamia	Tobramycin	Aztreonam, Cefepime, Cefotaxime, Ceftazidime, Ceftriaxone, Ciprofloxacin Piperacillin and Piperaciliin-Tazobactum, SXT	Metronidazole 500 mg IV QI, Piperacillin- Tazobactam 3.375/0.375 g IV QID Followed by Cefepime (2 g IV BID for 3 days) Ciprofloxacin (400 mg IV BID for 8 days) Imipenem (1 g IV for 7 days).	Complete recovery
Chun, et al., [70]	2009	Seoul, South Korea	53/M	Smoker. Previous injury that resulted in developing Pleural effusion. Chronic Empyema in lungs.	Bacteraemia (Cathether related)	Amikacin, Gentamicin, Tobramycin	Cefotaxime, Ceftazidime, Cefepime, Imipenem Piperacillin-Tazobactam, SXT	IV Moxifloxacin started. Changed to Imipenem -Cilastin (500 mg 6 h) and both catheters were removed.	Complete recovery
Kawamura, et al., [71]	2010	Japan	11/F	Metastatic neuroblastoma	Bacteraemia (Cathether related)	Amikacin, Cefepime, Cefozopran, Gentamicin, Piperacillin, Tobramycin	Ceftazidime, Ciprofloxaicn, Imipenem, Merpenem, Minocycline, SXT	eftazidime, removal of the catheter	Complete recovery
Mahmood, et al., [72]	2010	Kentucky, USA	30/M	Intravenous drug use, Hepatitis C, and posttraumatic stress disorder.	Acute infective Endocarditis	Aminoglycosides, Colistin, Cefazolin, Cefepime	Ceftazidime, Carbapenems, Fluoroquinolones, Piperacillin-Tazobactam, Tetracycline, SXT	Vancomycin, piperacillin-tazobactam changed to ceftriaxone,	Complete Recovery
Chotikanatis et al., [73]	2011	New York USA	10/F	Renal cortical necrosis and end-stage renal disease	Bacteraemia (Cathether related)	Aminoglycosides, Penicillin, Narrow-spectrum cephalosporins	Expanded-spectrum and broad-spectrum Cephalosporins, Aztreonam, Carbapenems, Piperacillin-Tazobactam, Quinolones Ticarcillin-Clavulanate,	14-day course of cefepime. 14-day course of Ceftazidime for second episode	Complete recovery
Kam et al. [74]	2011	Taiwan	93/M	Benign prostate hyperplasia with obstructive uropathy	Bactereamia associated with an ascending Urinary tract infection.	Cefepime, Cefoxitin, Cefuroxime, Cefoperazone, Ceftriaxone, Ciprofloxacin, Gentamicin, Levofloxacin, Netilmicin, Norfloxacin, Ofloxacin, Piperacillin, Piperacillin -Tazobactam, SXT, Tigecycline, Ticarcillin, Tobramycin	Cefoperazone – Sulbactam, Imipenem, Merpenem,	Cefoperazone/Sulbactam (1g/1g) for 6 h through IV. Followed by Imipenem 500 mg through IV for 6 h	Complete Recovery
Lang, et al., [26]	2011	India	65/M	NK cell lymphoma *Corynebacterium*	Line related sepsis	Aminoglycosides	Carbapenems, Cefatzidime, Ciprofloxacin, Piperacillin-Tazobactam, Trimethoprim	Piperacillin-Tazobactam, Gentamicin Patient had Hickman line removed Followed by Imipenem- Cilastatin, Teicoplanin	Complete recovery
Khan, et al., [75]	2012	India	4/F	None	Empyema	Gentamicin, Ceftazidime, Tetracycline, Meropenem	Cefoperazone-Sulbactam	Cefoperazone-sulbactam	Death (Septic Shock)
Hagiya, et al., [76]	2013	Japan	46/F	Organophosphorus poisoning	Bactereamia caused by bacterial translocation after organophosphorus poisoning	Cefepime, Gentamicin, Piperacillin	Amikacin, Aztreonam, Ceftazidime, Ciprofloxacin, Imipenem, Levofloxacin Meropenem, Minocycline, Ppiperacillin -Tazobactam, SXT	Ampicillin - Sulbactam Followed by Ppiperacillin -Tazobactam	Complete recovery
Ray & Lim [77],	2013	Singapore	14/F	No pre-existing medical condition *Chryseobacterium meningosepticum*	Keratitis associated with a Cosmetic contact lenses which caused a para-central corneal ulcer	Ampicillin, Ceftazidime, Ceftriaxone, Piperacillin.	Ciprofloxacin, Gentamicin	Cefazolin (50 mg/mL) and gentamicin (14 mg/mL) eyedrops Followed by Ciprofloxacin	Complete recovery
Camargo et al., [78]	2014	Brazil	Multiple (21 Cases)	N/A	Lung problems	Amikacin, Gentamicin Tobramycin	Cefotaxime, Cefepime, Imipenem, Levofloxacin	N/A	N/A
Khan and Krishnan [79]	2014	Australia	70/M	Type II Diabetes mellitus, Heart failure, COPD	Skin Infection	Amikacin, Amoxicillin, Gentamicin Tobramycin	Ciprofloxacin, SXT	Oral ciprofloxacin	Complete recovery
Lu and Huang [44]	2014–2022	Taiwan	60/M	2	Pneumonia	Amikacin, Gentamicin	Ceftazidime, Ciprofloxacin, Imipenem, Meropenem, Piperacillin- Tazobactam	Piperacillin- Tazobactam	Complete recovery
Lu and Huang [44]	2014–2022	Taiwan	80/F	3	Pneumonia	Amikacin, Cefepime, Ciprofloxacin Gentamicin	Ceftazidime, Imipenem, Meropenem, Piperacillin- Tazobactam	Cefepime	Complete recovery
Lu and Huang [44]	2014–2022	Taiwan	73/M	2	Pneumonia	Amikacin, Ciprofloxacin, Gentamicin	Ceftazidime, Cefepime, Imipenem, Meropenem, Piperacillin- Tazobactam	Cefepime	Complete recovery
Lu and Huang [44]	2014–2022	Taiwan	84/F	3	Pneumonia	Amikacin, Gentamicin	Ceftazidime, Cefepime, Imipenem, Meropenem, Piperacillin- Tazobactam	Ceftazidime	Complete recovery
Lu and Huang [44]	2014–2022	Taiwan	88/M	2	Pneumonia	Amikacin, Gentamicin	Ceftazidime, Cefepime	Imipenem	Complete recovery
Lu and Huang [44]	2014–2022	Taiwan	58/M	0	Pneumonia	Amikacin, Gentamicin	Ceftazidime, Cefepime, Imipenem, Meropenem, Piperacillin- Tazobactam	Ceftazidime	Complete recovery
Lu and Huang [44]	2014–2022	Taiwan	80/M	2	Pneumonia	Amikacin, Ciprofloxacin, Gentamicin	Ceftazidime, Cefepime, Imipenem, Meropenem, Piperacillin- Tazobactam	Ceftazidime	Complete recovery
Lu and Huang [44]	2014–2022	Taiwan	84/F	2	Pneumonia	Amikacin, Ciprofloxacin, Gentamicin	Ceftazidime, Cefepime, Imipenem, Meropenem, Piperacillin- Tazobactam	Imipenem	Complete recovery
Lu and Huang [44]	2014–2022	Taiwan	90/F	4	Pneumonia	N/A	N/A	Cefepime	Death
Lu and Huang [44]	2014–2022	Taiwan	37/F	2	Pneumonia	Amikacin, Ciprofloxacin, Gentamicin	Ceftazidime, Cefepime, Imipenem, Meropenem, Piperacillin- Tazobactam	Ceftazidime	Complete recovery
Lu and Huang [44]	2014–2022	Taiwan	87/F	2	Peritonitis	Amikacin, Ciprofloxacin, Gentamicin	Ceftazidime, Cefepime, Imipenem, Meropenem, Piperacillin- Tazobactam	Ceftibuten	Complete recovery
Lu and Huang [44]	2014–2022	Taiwan	55/M	3	Peritonitis	Amikacin, Cefepime, Gentamicin	Ceftazidime, Imipenem, Meropenem	Cefepime	Complete recovery
Lu and Huang [44]	2014–2022	Taiwan	89/F	2	Bacteraemia	Amikacin, Ciprofloxacin, Gentamicin	Ceftazidime, Cefepime, Imipenem, Meropenem, Piperacillin- Tazobactam	Ampicillin - Sulbactam	Complete recovery
Lu and Huang [44]	2014–2022	Taiwan	51/M	3	Bacteraemia	Amikacin, Ceftazidime, Cefepime, Gentamicin, Imipenem, Meropenem, Piperacillin- Tazobactam	Ciprofloxacin	Amoxicillin -Clavulanic acid	Complete recovery
Lu and Huang [44]	2014–2022	Taiwan	78/M	2	Bacteraemia	Amikacin, Cefepime, Ciprofloxacin, Gentamicin	Ceftazidime, Imipenem, Meropenem, Piperacillin- Tazobactam	Imipenem	Complete recovery
Lu and Huang [44]	2014–2022	Taiwan	89/M	1	Bacteraemia	Ceftazidime, Cefepime, Piperacillin- Tazobactam	Amikacin, Ciprofloxacin, Gentamicin	Ciprofloxacin	Death
Lu and Huang [44]	2014–2022	Taiwan	70/M	3	Liver abscess	Amikacin, Ciprofloxacin, Gentamicin	Ceftazidime, Cefepime, Imipenem, Meropenem, Piperacillin- Tazobactam	Imipenem	Complete recovery
Lu and Huang [44]	2014–2022	Taiwan	72/M	4	Urinary Tract Infection	Amikacin, Ciprofloxacin, Gentamicin	Ceftazidime, Cefepime, Imipenem, Meropenem, Piperacillin- Tazobactam	Ampicillin-Sulbactam	Complete recovery
Lu and Huang [44]	2014–2022	Taiwan	83/M	2	Urinary Tract Infection	Amikacin, Ciprofloxacin, Gentamicin	Ceftazidime, Cefepime, Imipenem, Meropenem, Piperacillin- Tazobactam	Ceftazidime	Complete recovery
Lu and Huang [44]	2014–2022	Taiwan	77/M	3	Urinary Tract Infection	Amikacin, Ceftazidime, Cefepime, Ciprofloxacin, Gentamicin, Meropenem, Piperacillin- Tazobactam	Imipenem	None	Complete recovery
Lu and Huang [44]	2014–2022	Taiwan	56/F	1	Urinary Tract Infection	Amikacin, Gentamicin	Ceftazidime, Cefepime, Ciprofloxacin, Imipenem, Meropenem, Piperacillin- Tazobactam	None	Complete recovery
Lu and Huang [44]	2014–2022	Taiwan	67/M	3	Urinary Tract Infection	Amikacin, Ciprofloxacin, Gentamicin	Ceftazidime, Cefepime, Imipenem, Meropenem, Piperacillin- Tazobactam	Ceftazidime	Complete recovery
Lu and Huang [44]	2014–2022	Taiwan	83/F	3	Urinary Tract Infection	Amikacin, Gentamicin	Ceftazidime, Cefepime, Ciprofloxacin, Imipenem, Meropenem, Piperacillin- Tazobactam	None	Complete recovery
Lu and Huang [44]	2014–2022	Taiwan	79/M	2	Urinary Tract Infection	Amikacin, Gentamicin	Ceftazidime, Cefepime, Ciprofloxacin, Imipenem, Meropenem, Piperacillin- Tazobactam	Meropenem	Death
Lu and Huang [44]	2014–2022	Taiwan	62/M	3	Urinary Tract Infection	Amikacin, Gentamicin	Ceftazidime, Cefepime, Ciprofloxacin Imipenem, Meropenem, Piperacillin- Tazobactam	Ceftazidime	Complete recovery
Lu and Huang [44]	2014–2022	Taiwan	71/F	2	Urinary Tract Infection	Amikacin, Ciprofloxacin, Gentamicin	Ceftazidime, Cefepime, Imipenem, Meropenem, Piperacillin- Tazobactam	Ceftazidime	Complete recovery
Bilgin, et al., [80]	2015	Canada	68/F	B cell acute lymphocytic leukaemia	Bactereamia associated with pneumonia	Amikacin, Ampicillin-Sulbactam, Gentamicin, Ciprofloxacin, Colistin	Expanded- and broad-spectrum Cephalosporins, Carbapenems, Piperacillin-Tazobactam	Piperacillin-Tazobactam −4.5 g/6 h, Ciprofloxacin, 400 mg/12 h	Complete recovery
Sharma et al. [81]	2015	India	Neonate/M	Neonate	Umbilical infection	Ceftazidime, Piperacillin -Tazobactum, SXT	Amikacin, Gentamicin, Imipenem, Meropenem, Piperacillin -Tazobactum, Tobramycin	Meropenem, Reicoplanin and Fluconazole	Complete Recovery
Patel, et al., [82]	2019	USA	49/F	Intravenous drug abuse Vertebral osteomyelitis	Septic pulmonary embolism (catheter-related infection)	All aminoglycosides	Ppiperacillin-Tazobactam	IV Piperacillin- Tazobactam. Catheter removal.	Complete Recovery
Yildiz, et al., [83]	2019	Turkey	52/F	Thrombocytopenic purpura (immuno-suppressive therap), Diabetes mellitus.	Pneumonia with lung cavities formation	Amikacin, Cefepime, Ciprofloxacin, Colistin, Gentamicin, Netilmicin.	Ceftazidime, Imipenem, Ppiperacillin Ppiperacillin-Tazobactam	IV piperacillin-tazobactam (4.5 g does 3 times/day) and oral clarithromycin (500 mg 2 times/day)	Complete recovery.
Smits, et al., [84]	2020	Germany	60/F	None	Infection of the orbital after a cat scratch 34 months earlier	N/A	N/A	Ppiperacillin - Tazobactam 3 × 4.5 g/d IV for 10 days.	Complete recovery
Deb, et al. [85]	2020	India	67/M	None Post cataract surgery	Post-operative Endophthalmitis	N/A	Ceftazidime, Ceftriaxone, Cefoperazone -Sulbactam, Chloramphenicol, Levofloxacin, Meropenem.	Started on intravitreal vancomycin (1 mg/0.1 mL) and ceftazidime (2.25 mg/0.1 mL). Followed by IV Cciprofloxacin 200 mg twice a day, topical moxifloxacin drops hourly, Followed by Ceftazidime (2.25 mg/0.1 mL) injection	Complete recovery
Artan et al., [86]	2020	Turkey	60/M	end-stage kidney disease, Diabeties	Peritonitis	Aminoglycosides, Colymicin a	Quinolones, Ceftazidime, SXT	1 g of cefazol and 1 g of ceftazidim daily Added Oral Ciprofloxacin 500 mg/day	Complete recovery
Ertan and Yılmaz [87]	2020	Turkey	4 month old/F	Congenital Heart Disease *Klebsiella pneumonia*e	Sepsis	N/A	N/A	Cefepime	Death
Perumal et al., [88]	2020	India	57/M	HIV Positive *Enterococcus faecium*	Pleural Effusion	Aminoglycosides, Fluroquinolnes	Cefaperazone-Sulbactam, Doripenem, Imipenem, Meropenem Piperacillin-Tazobactam	IV Cefoperazone-sulbactam	Complete recovery
Peters et al., [89]*Stenotrophomonas maltophilia*	2020	USA	62/F	Hypertension, COPD	Necrotizing pneumonia	N/A	Levofloxacin, SXT	Cefepime and Vancomycin Followed by Ampicillin-Sulbactam	Complete recovery
Højgaard et al. [43]	2002–2022	Demark	Multiple (59 Cases – 35 Male/24 Fenale)	Multiple (bone marrow transplant recipient; cardiovascular diseases, chronic obstructive pulmonary disease; congenital syndromes; connective tissue disease; cystic fibrosis; Diabetes mellitus; hematologic malignancies; interstitial lung disease; liver disease; neuromuscular disease; primary ciliary dyskinesia; renal dysfunction; solid cancer; solid organ transplant recipient)	Infections	Colistin, Gentamicin, Tobramycin	Ceftazidime, Ciprofloxacin Imipenem, Meropenem, Piperacillin-Tazobactam	Fluoroquinolones Meropenem	Not determined
Singh et al., [90]	2022	India	29/F	Breast Cancer	Sepsis	Amikacin, Colistin	Aztreonam, Ceftazidime, Ciprofloxacin, Meropenem, Levofloxacin, Piperacillin -Tazobactum	Meropenem, Amphotericin B, Teicoplani	Death
Lall et al. [91]	2023	India	35/M	Adenocarcinoma	Pleural Effusion	Gentamicin	Ceftazidime, Cefoperazone Sulbactam, Ciprofloxacin, Levofloxacin, Piperacillin- Tazobactam	Ciprofloxacin 500 mg	Complete recovery
Agarwal et al. [46]	2023	India	New Born/M	None	Bacteraemia	Amikacin, Ciprofloxacin, Colistin, Gentamicin,	Carbepenems, Cephalosporins, Piperacillin–Tazobactam	Ampicillin and Gentamcin	Death
Alam et al., [92]	2023	India	Neonate	Pneumonia	Sepsis	Amikacin, Cefepime, Colistin, Piperacillin- Tazobactam	Ceftazidime, Cefoperazone-Sulbactam, Ciprofloxacin, Levofloxacin Meropenem	Ciprofloxacin, Meropenem	Complete recovery
Backman et al., [93]	2023	USA	M/35	Membrano-proliferative glomerulonephritis	Bacteraemia	N/A	N/A	N/A	Complete recovery
Backman et al., [93]	2023	USA	M/50	Diabeties	Bacteraemia	N/A	N/A	N/A	Complete recovery
Peykov et al., [55]	2024	Bulgaria	65/F	None	Bronchitis	Colistin, Gentamicin	Amikacin Imipenem, Meropenem, Tobramycin.	Oral Levofloxacin, starting 500 mg twice daily for five days, followed by 500 mg once daily for ten days.	Complete recovery
Jaya et al., [94]	2025	India	16 month old/F	Eczema	Axillary Abscess	Ceftazidime, Piperacillin	Amikacin, Cefoperazone, Ciprofloxacin, Gentamicin, Imipenem, Meropenem, Netilmicin, Tetracycline, SXT	Inintravenous (IV) cloxacillin 200 mg/kg/d and IV clindamycin 40 mg/kg/d Followed by IV clindamycin was ceased and IV ceftriaxone 100 mg/kg/d Followed by oral flucloxacillin Followed by oral ciprofloxacin 40 mg/kg/d	Complete recovery
Scaglione et al., [95]	2025	Italy	61/M	Hypertension, Stroke, Epilepsy, End Stage Kidney Disease	Septic shock (cathether related)	Aminoglycosides	Cephalosporins, Fluoroquinolones, Piperacillin–Tazobactam	Meropenem and Vancomycin Followed by Piperacillin–Tazobactam	Complete recovery
Gordon and Marin [47]	2025	USA	78/F	Smoker, Stroke, Peripheral Artery Disease, Type 2 Diabetes mellitus, Hypertension	Pneumonia	Gentamicin	N/A	Levofloxacin and Minocycline	Death

M − Male, F- Female, N/A – Not Available, Lu and Huang did not list Co-morbidities, only giving the number of them, however they say that they were a combination of “cardiovascular diseases, malignancies, diabetes mellitus, cerebrovascular diseases, chronic kidney disease or end-stage renal disease, chronic obstructive pulmonary disease, autoimmune rheumatic diseases, and hepatitis B or C”.

**Table 3:** Incidences of *D. lacustris* infection from 2012 to 2024– Main characteristics of the case reports.

Reference	Year	Location	Age/sex	Pre-existing medical condition	Type of infection	Antibiotic resistance	Antibiotic Susceptibility	Treatment	Patient outcome
Shin, et al., [96]	2012	Germany	66/M	Alcoholic and chain smoker.	Empyema	Gentamicin	Amikacin, Aztreonam, Ceftriaxone, Cefepime, Ceftazidime, Imipenem, Levofloxacin	Imipenem	Complete Recovery
Shin, et al., [96]	2012	Germany	70/M	Benign prostate hypertrophy and Angina pectoris. Patient went to Hospital with a renal injury.	Unknown	Gentamicin	Amikacin, Aztreonam, Ceftriaxone, Cefepime, Ceftazidime, Imipenem, Levofloxacin	No antibiotic Treatment	Complete recovery
Shin, et al., [96]	2012	Germany	40/M	Hepatocellular carcinoma	Unknown	Ceftriaxone, Cefepime, Ceftazidime, Amikacin, Aztreonam and Gentamicin	Imipenem, Levofloxacin	Cefotaxime	Discharged in a grave condition
Shin, et al., [96]	2012	Germany	67/M	COPD, Myocardial infarction.	Unknown	Amikacin, Cefepime, Gentamicin	Aztreonam, Ceftriaxone, Ceftazidime, Imipenem, Levofloxacin	No antibiotic Treatment	Complete recovery
Sohn & Baek [97],	2015	South Korea	67/M	Diabetes mellitus, Hypertension. Pheochromocytoma	Bactereamia caused by peripheral intravenous catheter which led to septicaemia	Aminoglycosides, Ciprofloxacin, Ticarcillin,SXT	Aztreonam, Cefotaxime, Ceftazidime, Piperacillin Carbapenems	Iv Cefazolin. Followed by Piperacillin -Tazobactam	Complete recovery
Sohn, et al., [98]	2015	South Korea	70/M	Patient was taking anti-hypertensive pills and anti-diabetic pills.	Keratitis	Amikacin, Gentamicin	Aztreonam, Cefepime, Ceftazidime, Ciprofloxacin, Imipenem, Meropenem, Piperacillin -Tazobactam, Ticarcillin -Clavulanic acid	Fortified topical Ofloxacin, Voriconazole, Gentamicin Followed by topical Ciprofloxacin, systemic Ceftazidime	Evisceration (Eye removal)
Cousillas et al., [99]	2024	Spain	78/M	Chronic Kidney Disease, Hypertension, Dyslipidemia, and Type 2 Diabetes Mellitus	Peritoneal infections	N/A	N/A	Intraperitoneal Vancomycin 2 g and gentamicin 80 mg, Meropenem, Cathather Removal	Complete recovery

**Table 4:** Incidences of *D. tsuruhatensis* infection from 2012 to 2024– Main characteristics of the case reports.

References	Year	Location	Age/Sex	Pre-existing Medical Condition	Type of infection	Antibiotic resistance	Antibiotic Susceptibility	Treatment	Patient outcome
Preiswerk, et al., [100]	2012	Switzerland	53/F	Idiopathic pulmonary hypertension	Bactereamia (Catheter-related)	Amikacin, Ampicillin, Cephalothin, Cefuroxime, Colistin Gentamicin, Tobramycin	Amoxicillin–Clavulanate, Ceftriaxone, Ceftazidime, Cefotaxime, Cefepime, Ciprofloxacin, Ertapenem Levofloxacin, Imipenem, Meropenem, Piperacillin–Tazobactam,	Oral Ciprofloxacin	Complete recovery
Ranc, et al., [27]	2008	Marseille, France	77/M	Liver cancer, colic adenocarcinoma	Considered by physicians as colonization	N/A	N/A	N/A	Complete recovery
Ranc, et al., [27]	2009	Marseille, France	70/F	Immunocompromised	unknown	N/A	N/A	N/A	Complete recovery
Ranc, et al., [27]	2010	Marseille, France	59/F	Alcoholism which led to chronic end-stage renal failure	Catheter-related bloodstream infection; (Septicaemia)	N/A	N/A	Gentamicin Piperacillin-Tazobactam	Complete recovery
Tabak, et al., [101]	2010	Turkey	53/F	Metastatic breast cancer	Bactereamia (Port-related)	N/A	Third generation cephalosporins, Cefepime, Quinolones, and beta-lactamase inhibitors	Ceftriaxone 1 g/day for 14 days	Complete recovery
Ranc, et al., [27]	2010	Marseille, France	6/M	Cystic fibrosis	unknown	N/A	N/A	N/A	Complete recovery
Ranc, et al., [27]	2013	Marseille, France	42/M	Chronic renal failure, alcoholic Hepatitis C	unknown	N/A	N/A	N/A	Complete recovery
Ranc, et al., [27]	2014	Marseille, France	13/F	Liver transplant	Post-transplant fever;	N/A	N/A	Piperacillin -Tazobactam	Complete recovery
Ranc, et al., [27]	2015	Marseille, France	82/M	Haemodialysis, vascular dementia	Catheter-related bloodstream infection; (Septicaemia)	N/A	N/A	Ceftazidime	Complete recovery
Ranc, et al., [27]	2015	Marseille, France	47/M	Kidney transplant	Fever (Bactereamia)	N/A	N/A	N/A	Complete recovery
Ranc, et al., [27]	2015	Marseille, France	6 months/F	Preterm birth	Ventilator-associated pneumonia;	Amoxicillin, Amoxicillin- Clavulanate	Ceftriaxone, Ertapenem, Imipenem, Ofloxacin	Ceftazidime, Followed by Imipenem, Vancomycin Amikacin Followed by Tobramycin	Death
Cheng et al., [49]	2019	China	91/M	N/A	Rrespiratory failure	Ampicillin, Cefazolin, Amikacin, Ampicillin, Cefazolin,Gentamicin, Netilmicin, Streptomycin, Tetracycline, SXT, Tobramycin	Azithromycin, Aztreonam, Cefoxitin, Ceftriaxone, Ceftazidime, Cefepime, Chloramphenicol, Ciprofloxacin, Florfenicol, Levofloxacin, Imipenem	N/A	N/A
Cho et al., [48]	2019	Korea	65/M	Stomach cancer (Total gastrectomy)	Fever	Amikacin, Ceftazidime, Cefepime, Ceftriaxone, Levofloxacin, Meropenem	Minocycline, Piperacillin-Tazobactam	Ciprofloxacin Followed by Piperacillin-Tazobactam	Complete recovery

**Table 5:** Incidences of *Delftia sp* infection from 2015 to 2024– Main characteristics of the case reports.

References	Year	Location	Age/Sex	Pre-existing Medical Condition	Type of infection	Antibiotic resistance	Antibiotic Susceptibility	Treatment	Patient outcome
Kang, et al., [48] *Delftia* sp. 670	2015	China	33/M	HIV, Pulmonary infection, respiratory failure and a spinal deformity	Bactereamia associated with pneumonia	Amikacin, Ampicilin, Cefuroxime-sodium, Cefazolin	Aztreonam, Cefepime, Cefotetan, Ceftazidime, Ceftriaxone, Ciprofloxacin, Levofloxacin, Imipenem, Meropenem, Piperacillin	N/A	Death after 10 days
(Böttger, et al., [102])	2020	Germany	23/F	None	Chronic Wound Infection after Wisdom Tooth Extraction 6 months earlier	N/A	N/A	N/A	Complete recovery

## 3 Delftia Infections

### 3.1 *Delftia* spp instances of infection

All recorded instances of infection with *Delftia* spp in humans from the scientific and medical literature can be found in Table 2 (*D. acidovorans)*, 3 (*D. lacustris*), 4 (*D. tsuruhatensis*) and 5 (unidentified *Delftia* sp.). No instances of infection have been reported with the other three recognised Delftia species. These tables show the date (year) of infection (if unavailable publication year was used instead), geographic location (country was used as in most cases no smaller geographic information was given) of infection, information on the patient(s) [age, gender, prior health conditions], infection type (bacteraemia, meningitis, etc) caused by the *Delftia* sp infection, antibiotic susceptibility testing undertaken (both susceptibility and resistance were recorded where available), handling of infection (this focused primarily on the antibiotic treatments used and removal of any indwelling devices) and patient outcome (recovery, death, etc.).

Table 2, Table 3, Table 4, Table 5 show 175 separate instances of infection caused by *Delftia* spp. that were found in literature sources. Most of these infections were caused by *Delftia acidovorans* (153 instances – 87.4 %), other infections were due to *Delftia lacustris* (7 instances – 4 %), *Delftia tsuruhatensis* (13 instance – 7.4 %) and *Delftia* sp (2 instance – 1 %.). Many of the cases of *Delftia acidovorans* come from two large scale retrospective studies carried out by Højgaard et al. (2002–2022) [43] and Lu and Huang (2014–2022) [44].

132 (75.9 %) of the patients were found to have either one or more prior health conditions (either chronic or acute). Seventeen (9.8 %) of the patients were found to have no prior health conditions and for 25 patients (14.4 %) no information was provided/available. Sixty-Seven patients had one prior medical condition, and all other patients had two or more. By far the biggest pre-existing medical conditions were various different types of cancer (in 46 patients – 26.4 %), cardiovascular disease (in 33 patients – 19 %), Diabetes mellitus (in 13 patients – 7.5 %), HIV (in 3 patients – 1.7 %) and intravenous drug use (in 3 patients – 4.3 %). A full list of pre-existing medical conditions can be seen in Table 2, Table 3, Table 4, Table 5 Multiple different infection types were caused by the different Delftia species including pneumonia, bacteraemia, sepsis/septic shock, endocarditis and ocular infections.

The majority of cases were successfully treated with antibiotics and completed full recovery, 10 cases (14.5 %) either had no antibiotic usage or no recorded antibiotic usage and had a complete recovery, 11 patients (11.6 %) died, one patient with keratitis had to have the infected eye eviscerated. Patients who died due to *Delftia* spp. infection (Sepsis, pneumonia, empyema and endocarditis) had at least one pre-existing medical condition bar one patient (discussed below). No pseudo-outbreaks have been so far found associated with *Delftia* spp.

### 3.2 Mortality associated with *Delftia* spp. infection

Eleven instances of death associated with *Delftia* spp. infection have been recorded in the literature. Nine cases were linked to *D. acidovorans* (Table 2), one to *D. tsuruhatensis* (Table 4) and one to an unidentified *Delftia* spp (Table 5). No deaths have been associated with *D. lacustris* (Table 3). The first instance of death was recorded by Horowitz, et al., the patient in this case was an intravenous drug user and alcoholic [25]. The patient a 42-year-old female contacted *D. acidovorans* related endocarditis and later died. A second death was recorded in a 4-year-old female patient. She died of *D. acidovorans* related septic shock [45]. The *D. acidovorans* infection was related to an indwelling device*.* The third death was associated with *D. acidovorans* was a a case of sepsis in a 4 month female. She suffered from congenital heart disease. The fourth case of death assocatied with *D. acidovorans* was sepsis in a 29 year old female paitent undergoing chemotherapy for breast cancer. The fifth case was in a newborn male who contracted bacteremia [46]. The sixth case of death associated with *D.acidovroans* was in a 78 year old woman who died of pneumonia. She has multiple underlying conditions (Peripheral Artery Disease, Type 2 Diabetes, Hypertension). The infection was catheter related [47]. Three death were found in the retrospective study carried out by Lu and Huang. All three patients had cancer and death due to UTI, bacteremia and pneumonia [44]. Several deaths occurred in the study carried out by Højgaard et al. however these could not be linked directly to *Delftia acidovornas* infection [43]. Ranc et al., recorded a death due to ventilator-associated pneumonia; assoicated with *D. tsuruhatensis* in a 6 month old female who had been born prematurely [27]. A case of death due to severe pneumonia has also been linked to *Delftia* sp 670 in a 33 year old HIV Positive male [48].

### 3.3 Treatment of *Delftia* spp. infections

*Delftia* species have been shown to be resistant to many of the major antibiotic groups [[49], [50], [51]] (Cho et al., 2021; Cheng et al., 2021). This can cause complications when attempting to treat infections with this genus. Antibiotic treatment on *Delftia* spp. must be based on in vitro susceptibility testing carried on the infectious isolates. A wide array of antibiotics has been used to treat *Delftia* spp. infections. In most cases, *Delftia* spp. are susceptible to ceftazidime, ciprofloxacin and imipenem (based on Antibiotic Susceptibility testing data from the literature see Table 2, Table 3, Table 4, Table 5). Multiple isolates showed resistance to gentamicin so this should be avoided in treating Delftia infections. These results broadly agreed with the results of a major retrospective study carried out by Højgaard et al., on *D. acidovorans* that examined 18 years' worth of infections at a tertiary referral hospital in Copenhagen, Denmark, and the results of another retrospective study carried out by Lu and Huang that examined eight years' worth of infections in a hospital in a Taiwanese hospital [43,44]. Højgaard et al., found that meropenem or ceftazidime were the most effective antibiotics against *D. acidovorans* with ciprofloxacin and imipenem also proving to be effective and that the majority of isolates tested were resistant to gentamicin [43]*.* Lu and Huang found similar results with most isolates susceptible to meropenem and ceftazidime along with piperacillin-tazobactam and resistant to gentamicin. However, a major difference can be seen when it comes to ciprofloxacin where they found most of the isolate's resistance to the antibiotic [44].

The genetic basis for antibiotic resistance in *Delftia* sp Genome analysis of a *D. tsuruhatensis* pathogenic isolate identified 17 resistance genes including aminocyclitols (*aadA2b*), fluoroquinolone (*aac(6′)-Ib-cr*), aminoglycosides (*aph(3′')-Ib, aph(6)-Id aac(6′)-Ib3, aac(6′)-Ib-cr,* and *aadA2b*), folate pathway antagonist (*sul1, sul2*, and *dfrA16*), tetracycline (*tet(G)*), and phenicol (*cmx* and *floR*) [39], however resistance was not found in all strains supporting the conclusion of these strains evolving and adapting via horizontal gene transfer and adaption. A strain of *D. tsuruhatensis* MR-6/3H from cow milk was found to be resistant to 19 antibiotics of 23 tested. This included all aminoglycosides, phenicols, to trimethoprim-sulfamethoxazole and most β-lactams. Comparative genomic analysis revealed putative antimicrobial resistance genes mostly associated with antibiotic efflux systems [40]. The emergence of resistant determinants in these Delftia species may thus have clinical relevance for this species.

### 3.4 Infection prevention

Infections caused by *Delftia* species, are sporadic but are medically significant as they are primarily healthcare-associated. Nosocomial cases are normally associated with environmental reservoirs and indwelling medical devices reflecting the ability of Delftia species to persist in aqueous environments and form biofilms [41,42]. Hospital water systems, and contaminated fluids or equipment have all been shown as potential sources of infection [52]. Due to this infection prevention in healthcare settings should prioritise water system management, strict aseptic technique during catheter insertion and maintenance, and the removal of unnecessary intravascular lines. Isolation of *Delftia* spp from sterile sites should not be dismissed as contamination, as delayed recognition may inhibit correct infection-control interventions.

Community-acquired *Delftia* infections are uncommon and normally occur in individuals that have underlying medical conditions. Prevention outside healthcare settings depends on more general control measures, such as good personal hygiene, proper wound care, and safe handling of water in home-care or outpatient medical settings, instead of organism-specific public health strategies.

### 3.5 Surveillance and epidemiology of delftia species

Epidemiological data on *Delftia* species is highly limited. The data that is available comes mostly from descriptions in medical case reports and form retrospective laboratory analyses (Table 2, Table 3, Table 4, Table 5) rather than from surveillance programmes. *Delftia* spp are not commonly included in national or international surveillance programmes. They are usually included with other non-fermenting Gram-negative bacteria, which may lead to underestimation of incidences and of clinical significance [27,53]. The use of MALDI-TOF MS and whole-genome sequencing (WGS) has significantly improved identification of non-fermenting Gram-negative bacteria including *Delftia* spp, aiding to more regular identification of clinically relevant isolates in recent years [[54], [55], [56]].

With no *Delftia*-specific surveillance frameworks, approaches that have been developed for other opportunistic non-fermenting Gram-negative bacteria such as *Pseudomonas aeruginosa*, *Acinetobacter baumannii*, and the *Burkholderia cepacia* complex maybe appropriate. These include laboratory-based surveillance linked to clinical data, separation of community-acquired and healthcare-associated infections, and environmental surveillance of hospital water systems and medical devices [57,58]. Such methodologies are particularly appropriate given *Delftia*'s environmental persistence.

## 4 Conclusions

Although *Delftia* species are not thought of as major human pathogens, the evidence indicates that they represent rare opportunistic causes of infection, with approximately 175 cases reported across the global over several decades. This support the view that *Delftia* spp. are uncommon, generally low-virulence organisms. The clinical outcomes in cases of Delftia infection are in most cases good with patients recovering completely. *Delftia* spp are usually susceptible to several different antimicrobial classes, including carbapenems and fluoroquinolones. This indicates that issues posed by *Delftia* spp infections should be dealt with in the context of surveillance of non-fermenting Gram-negative bacteria generally, rather than be seen as a concern in and of itself in infection control. Overall, the available evidence supports the view of *Delftia* as rare, (usually) treatable opportunistic pathogens.

## CRediT authorship contribution statement

**Michael P. Ryan:** Writing – review & editing, Writing – original draft, Investigation, Data curation, Conceptualization. **J. Tony Pembroke:** Writing – review & editing, Writing – original draft, Data curation, Conceptualization.

## Declaration of competing interest

The authors declare that they have no known competing financial interests or personal relationships that could have appeared to influence the work reported in this paper.
